# CONSORT statement adherence and risk of bias in randomized controlled trials on deep caries management: a meta-research

**DOI:** 10.1186/s12903-024-04417-0

**Published:** 2024-06-13

**Authors:** Rokaia Ahmed Elagami, Thais Marchezini Reis, Mohamed Ahmed Hassan, Tamara Kerber Tedesco, Mariana Minatel Braga, Fausto Medeiros Mendes, Maximiliano Sérgio Cenci, Marie-Charlotte Huysmans, Daniela Prócida Raggio

**Affiliations:** 1 Department of Orthodontics and Pediatric Dentistry, Faculty of Dentistry, University of São Paulo, São Paulo, Brazil; 2 Department of Stomatology, Faculty of Dentistry, University of São Paulo, São Paulo, Brazil; 3 Department of Dentistry, Radboud University Medical Center, Research Institute for Medical Innovation, Nijmegen, The Netherlands

**Keywords:** RoB 2 tool, Randomized clinical controlled trials, Dental caries management, CONSORT adherence, Pediatric dentistry

## Abstract

**Background:**

Recently, trials have supported changes in deep caries management. However, reporting might lack details, affecting interpretation and implementation. Thus, we aimed to evaluate the adherence to the CONSORT statement and the risk of bias of randomized controlled trials (RCTs) on deep caries management published in pediatric dental journals.

**Methods:**

We searched PubMed for RCTs in six pediatric dental journals between 2010 and 2022, focusing on deep caries lesion management. Adherence to the CONSORT guideline and the risk of bias were assessed using a modified tool with 19 items; each scored from 0 to 2 (maximum of 38 points), and the Cochrane risk-of-bias (RoB 2) tool. We performed descriptive and regression analyses (*α* = 5%).

**Results:**

We analyzed 127 RCTs. The mean (standard deviation) CONSORT adherence score was 21.1 (6.7). Notably, 96.1% of the studies received a score of 2 for the "intervention" item, whereas 83.5% scored 0 for the "estimated effect size”. The risk of bias assessment revealed that 40.2% of the RCTs were at high risk, 59% were at low risk, and 0.8% were at low risk. RCTs with a high risk of bias had lower CONSORT scores (*p*<0.001) than those with low or some concerns. RCTs published in journals without the endorsement of the CONSORT statement had lower scores than those in journals with the endorsement of the CONSORT statement. Older RCTs (6-10 years old and more than 10 years old) showed significantly lower CONSORT statement compliance than trials published recently within 5 years.

**Conclusion:**

Adherence to the CONSORT was relatively low among the investigated RCTs. Moreover, lower adherence to the CONSORT was associated with a higher risk of bias.

**Trial Registration:**

This study protocol was prospectively registered on the Open Science Framework - DOI (10.17605/OSF.IO/V6SYZ).

**Supplementary Information:**

The online version contains supplementary material available at 10.1186/s12903-024-04417-0.

## Background

Deep caries refers to caries lesions that penetrate the inner third of dentin, carrying a risk of exposing the pulp. Traditionally, deep caries management has centered on complete or nonselective caries removal. However, recent research results advocate techniques such as minimally invasive and biologically based approaches [1]. Due to the importance of this topic, many randomized controlled trials (RCTs) have been conducted to investigate the best management methods for deep caries lesions [2].

However, as wisely quoted by Professor Douglas Altman, "To maximize the benefit to society, you need to not just do research, but do it well" [3]. Therefore, it is imperative to employ properly designed and implemented methodologies to ensure the production of reliable scientific conclusions [4]. In dentistry, all decisions made by practitioners should be based on well-conducted and transparent research to provide effective and safe treatments [5] rather than relying solely on personal experiences or expert opinions [6].

According to the hierarchy of evidence, RCTs are considered the gold standard for assessing the impact of interventions in clinical care [7]. Therefore, they should be meticulously designed to prioritize transparency and impartiality [8]. Poorly designed RCTs have the potential to harm patients and lead to wasted research efforts. This may involve various stages of the study, including the formulation of the research question setting, methodological choices, accessibility of data, and quality of reporting [9]. As a result, the value of an RCT is primarily contingent upon its “internal validity,” which is achieved through proper methodological rigor and adherence to best practices [10].

Approximately 1.5 million articles are published annually in scientific journals [11]. Numerous initiatives have been undertaken to enhance research transparency and mitigate publication bias. These include compliance with reporting guidelines and the pre-registration of research protocols. To improve the quality of RCTs, the Consolidated Standards for Reporting of Trials (CONSORT statement) developed a checklist that consists of 37 items that delineate crucial data that a well-designed RCT should incorporate in its reporting [12]. To facilitate the assessment of compliance with CONSORT guidelines, Reis and colleagues developed an instrument aligning with the CONSORT items [13]. Moreover, a standardized tool for evaluating quality, known as the Cochrane Risk of Bias (RoB), was introduced in 2008 and was last updated in 2019 (RoB 2). When using the RoB 2 tool, bias is assessed in five distinct domains: selection bias, performance bias, detection bias, attrition bias, and reporting bias. These assessments are informed by answers to one or more signaling questions and result in judgments of “low risk of bias,” “some concerns,” or “high risk of bias” [14].

The developers of systematic reviews and clinical practice guidelines (CPGs), who incorporate clinical recommendations for pediatric dentists, should assess the internal validity and risk of bias in RCTs before utilizing their results. A proper evaluation of the reporting quality, methods, and potential biases in RCTs can enhance the validity of the resulting recommendations and the quality of care provided to patients [15]. Clinicians often encounter challenges when making treatment choices and selecting cost-effective procedures for managing deep caries lesions in pediatric dental patients. These challenges, which encompass factors such as the depth of caries and the affected tooth surfaces, can significantly influence the quality of care provided to pediatric patients. Therefore, it is imperative to have reliable sources of evidence that can guide clinical decision-making in this context [16]. With this context in mind, our objective was to evaluate adherence to the CONSORT statement and assess the risk of bias assessment of RCTs related to deep caries management published from 2010–2022 in pediatric dental journals. Our hypothesis was that RCTs published earlier would demonstrate decreased adherence to the CONSORT guidelines.

## Methods

### Protocol and registration

This research constitutes a meta-research project. The study protocol was prospectively registered on the Open Science Framework platform (10.17605/OSF.IO/V6SYZ).

### Information sources and search strategy

MEDLINE (PubMed) was chosen as the primary electronic database for identifying eligible studies, given that all the target journals are indexed there. To conduct our systematic search in accordance with best practices, we employed a MEDLINE search strategy using terms related to connections with randomized controlled trials and six pediatric dental journals. Boolean operators such as “AND” and “OR” were used to facilitate a comprehensive search (Supplementary file 1). We selected six representative pediatric dentistry journals, all of which were indexed in the Web of Science. The selected journals (2022 impact factors) were International Journal of Paediatric Dentistry (IF=3.8), Pediatric Dentistry (IF= 1.6), Journal of Clinical Pediatric Dentistry (IF=1.3), European Archives of Paediatric Dentistry (IF=2.2), Journal of Dentistry for Children (IF=0.8) and European Journal of Paediatric Dentistry (IF=3.6). Our search was restricted to articles published between 2010 and 2022, aligning with the last update of the CONSORT statement in 2010.

### Study selection and eligibility criteria

We included randomized controlled trials that compared two or more restorative treatments, techniques, or endodontic procedures for deep caries lesions in pediatric dentistry (up to 18 years old). These RCTs had to be published in one of the aforementioned six selected journals between 2010 and 2022. In vitro studies, observational studies (cohort, cross-sectional, and survey), prevention RCTs, behavior management studies, pain management studies, micro-invasive treatment studies, non-invasive treatment studies, studies not related to caries management, orthodontic studies, educational RCTs, studies on adult populations, or sedation studies were not eligible. For publications with no accessible content, we made three weekly email attempts to contact the authors. We excluded the publication from our study when we did not receive a response after these attempts. Two reviewers (RAE, TMR) independently screened the titles and abstracts to identify eligible articles. In cases where this information was insufficient, the reviewers read the full article.

### Data extraction

Two independent reviewers (RAE, TMR) extracted the data in tables structured in Excel spreadsheets created specifically for this research. Any uncertainties or queries that arose at any stage were addressed by consulting a third reviewer (DPR), who is considered an expert in the field. For the publications included in our analysis, we systematically collected the following information: title, journal's name, journal's impact factor (as per Journal Citation Reports - 2022), year of publication, author's e-mail, corresponding author’s country income (according to the World Bank Group), presence of the term "randomized trial" in the title, trial design, duration of follow-up, allocation ratio, sample size, whether sample size calculation was based on the primary outcome or not, method of randomization, allocation concealment, blinding, number of arms and interventions, protocol registration number and platform (if available), funding source (for-profit, non-profit, not reported or unclear), authors' declarations of using the CONSORT reporting checklist, and disclosure of authors' conflicts of interest. Additionally, when protocol registration was reported, we extracted data on the registry date and study start date to determine whether it was retrospective (i.e., the registration occurred after the enrollment of the first participant) or prospective (i.e., registration preceded the enrolment of the first participant). Information about the Principal Investigator (typically the first or last author) was collected, including their h-index (until August 2023). ​​In addition, we extracted the number of citations of each included RCT from the Web of Science.

### Evaluation of reporting quality and risk of bias

Randomized controlled trial transparency and reporting quality were evaluated by the same two independent reviewers (RAE, TMR), who assessed compliance with the CONSORT criteria. This assessment was based on the evaluation tool originally developed by Reis and colleagues [13] to assess reporting completeness. We slightly modified the original tool, including two new items (the title, abstract, and funding). We also adjusted the points related to registration and protocol by introducing a new scoring point. A score of 0 indicates “The authors describe that the study was registered but fail to provide the registration number and/or the provided number does not correspond to the study”. Additionally, we modified score 1 to “The registry number was not disclosed in the paper but was obtained through communication with the corresponding author”. Our modified tool comprises a total of 19 main items, including some subdivided items adapted from the CONSORT checklist (Supplementary file 2). Each item is scored on a scale from 0 to 2, with 0 indicating no description, 1 denoting poor description, and 2 indicating adequate description. To assess the overall quality of each article included, we calculated a cumulative score by summing the scores of all 19 items. A trial that provides complete and clear reports (score 2) for all items would attain the maximum possible score of 38.

In assessing the risk of bias for the included studies, two independent reviewers (RAE, TMR) performed the risk of bias assessment, and any discrepancies were resolved through consultation with a third expert reviewer (MAH). We conducted the risk of bias assessment using the RoB 2 as recommended by the Cochrane Handbook Systematic Reviews of Interventions. The RoB 2 tool (available on the riskofbiasinfo.org website) comprises five specific domains: bias arising from the randomization process, bias due to deviations from intended interventions, bias due to missing outcome data, bias in the measurement of the outcome, and bias in the selection of the reported results [14]. Each domain includes signaling questions designed to assist assessors in evaluating the risk of bias and can be categorized as follows: Yes/Probably yes/No/Probably no/No information. For the overall risk of bias judgment, three possibilities exist: low risk of bias (i.e., the study demonstrates a low risk of bias across all domains), some concerns (i.e., the study exhibits some concerns in at least one domain, without a high risk of bias in any domain), or high risk of bias (i.e., the study reports a high risk of bias in at least one domain or demonstrates some concerns in multiple domains). Furthermore, we employed a distinct version of the RoB 2 tool specifically designed for crossover RCTs using the March 18^th^, 2021 version.

### Statistical analysis and data synthesis

To assess the agreement between reviewers, an inter-rater reliability Cohen's kappa test was conducted for 10% of the included studies [17]. We conducted a descriptive analysis of the characteristics of the included studies. Qualitative variables were summarized using frequency distributions, while quantitative variables were described by means and standard deviations.

In the regression analysis, univariate exploratory analyses were performed, followed by constructing multiple models guided by the variables' significance. Only variables with *p*<0.05 were retained in the final models. The normality assumption of the CONSORT adherence scores was first checked through the Shapiro-Francia test. Since normality was observed, we conducted univariate and multiple linear regression analyses to assess the associations among explanatory variables, such as years since publication, RoB 2 overall assessment, journal endorsement of CONSORT, study design, funding, protocol registration, country income, declaration of adherence to CONSORT, H-index first author, impact factor for 2022, and CONSORT adherence scores (outcome variable).

For the logistic regression, the RoB overall assessment was considered as a dichotomous outcome variable (high risk of bias vs. low risk of bias or with some concerns). The associations between this outcome and the aforementioned variables were assessed, including CONSORT scores, country income, years since publication, study design, funding, protocol registration, journal endorsement of CONSORT, declaration of adherence to CONSORT, H-index for first author, and journal impact factor 2022. The odds ratios (ORs) and 95% confidence intervals (95% CIs) were calculated. Statistical significance was determined when *p*<0.05. We used Stata/SE version 15.0 (StataCorp, College Station, TX) to perform all the statistical analyses.

## Results

### Characteristics of the included trials

**Fig. 1:**
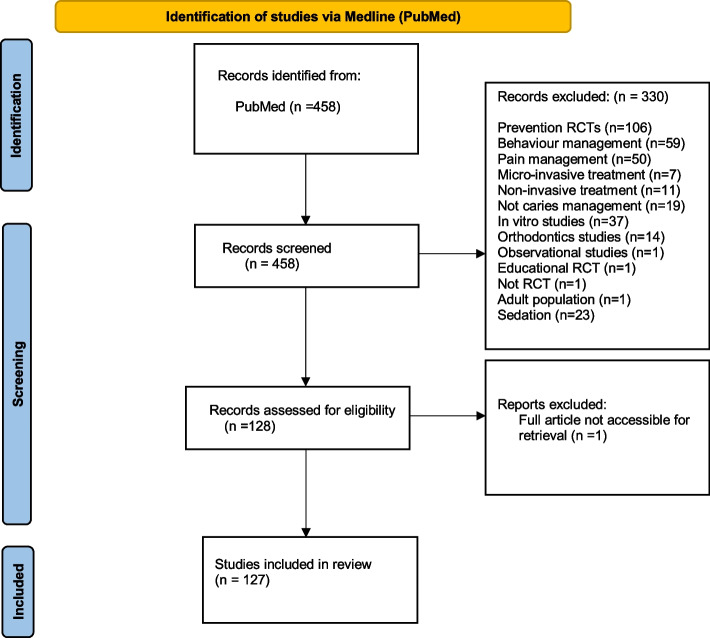
Flowchart of the study

The Cohen's kappa coefficient was 0.90, revealing almost perfect agreement between the reviewers. Out of the initial 458 documents, 127 RCTs were included in the analysis (Fig. 1). Table 1 shows the general characteristics of the 127 selected RCTs. When examining the distribution of deep caries management RCTs published between 2010 and 2022 in the targeted journals, we observed that the majority were published in the “Pediatric Dentistry” journal (30.7%), followed by the “Journal of Clinical Pediatric Dentistry” (20.5%). Notably, 62.2% of the articles did not explicitly specify their study design within their methodology section. Of the 72 RCTs that provided information about sample size estimation, 37 were unclear regarding whether this calculation was based on the primary outcome. Four journals endorsed the CONSORT statement in the author’s guidelines, namely, the “International Journal of Paediatric Dentistry”, “Pediatric Dentistry”, “European Archives of Paediatric Dentistry”, and “Journal of Dentistry for Children”. Our analyses showed that 55.9% of the studies failed to disclose the funding sources in the reports. Most studies (74%) did not report the protocol registration, while only six studies were registered prospectively, and 27 were registered retrospectively. All target journals endorsed the declaration of conflict-of-interest statements in the instructions of the authors except for the "Journal of Clinical Pediatric Dentistry". However, despite these guidelines, 67.7% of the articles did not declare a conflict of interest statement in the published article. Supplementary file 3 illustrates the World Bank country income classification with the representative countries for each category and the respective percentages of articles published between 2010 and 2022 in the target journals. Among these, India (20.5%) and Turkey (16.5%) were the countries with the greatest number of publications in the included journals.

**Table 1:** Characteristics of the 127 included randomised controlled trials

Characteristics	All RCTs (127)
**Journal, n (%)**	
International Journal of Paediatric Dentistry	25 (19.7)
European Journal of Paediatric Dentistry	11 (8.7)
Pediatric Dentistry	39 (30.7)
Journal of Clinical Pediatric Dentistry	26 (20.5)
European Archives of Paediatric Dentistry	22 (17.3)
Journal of Dentistry for Children	4 (3.1)
**Year Since Publication, n (%)**	
≤5 years	45 (35.4)
6-10 years	49 (38.6)
>10 years	33 (26)
**Study Design, n (%)**	
Parallel/factorial	21 (16.5)
Split mouth/crossover	27 (21.3)
Unclear	79 (62.2)
**Protocol registration, n (%)**	
Prospective registration	6 (4.7)
Retrospective registration	27 (21.3)
No registry	94 (74)
**Funding, n (%)**	
Non-Profit funding	40 (31.5)
No funding	7 (5.5)
For-Profit funding	9 (7.1)
Unclear	71 (55.9)
**Declaration of following CONSORT checklist, n (%)**	
No	108 (85)
Yes	19 (15)
**Declared “Randomised clinical trial” in the title, n (%)**	
No	64 (50.4)
Yes	63 (49.6)
**Declared conflict of interest statement, n (%)**	
No	86 (67.7)
Yes	41 (32.3)
**Sample size estimation**	
No	55 (43.3)
Yes	72 (56.7)
**Follow up Period (Months)**	
Min-Max	1-48
Mean (SD^a^)	16.2 (11.3)
**H- Index first author**	
Min-Max	0-35
Mean (SD)	6 (5.8)
**H- Index last author**	
Min-Max	0-58
Mean (SD)	10.5 (10.5)

^a^*SD* Standard Deviation

### Adherence to the CONSORT statement

**Fig. 2:**
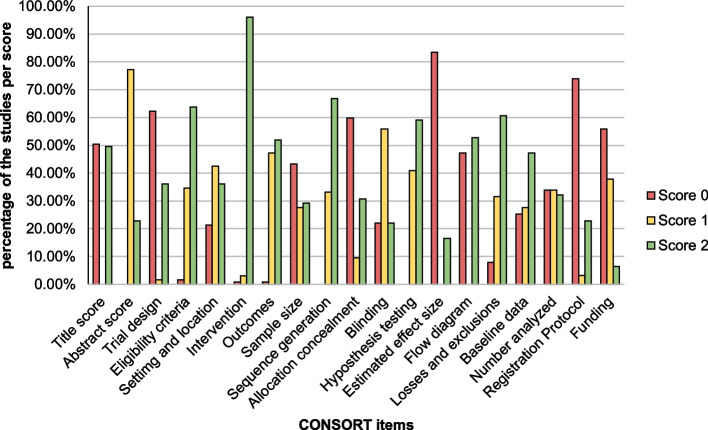
Represent the percentage of CONSORT compliance for each item for the included studies

The studies included in this review had a mean (standard deviation – SD) CONSORT adherence score of 21.1 (± 6.7). Figure 2 provides a detailed breakdown of CONSORT compliance for each item for the studies included. Among the total RCTs reviewed, 60 (47.2%) did not provide a flow chart and received a score of 0 for this item. For the “Abstract” item, 77.2% of the studies provided insufficient information, indicating poor reporting of the methodological steps. The evaluation of the "Sequence generation" and "Hypothesis testing" items revealed that both items demonstrated inadequate reporting at rates of 33.1% and 40.9%, respectively. The items that exhibited the most significant shortcomings, with a score of 0, were “Estimated effect size” (83.5%), “Protocol registration” (74%), and “Trial design” (62.2%). Conversely, items that were generally adequately reported (i.e., receiving a maximum score of 2) included “Description of interventions” (96.1%), followed by “Sequence of generation” (66.9%), and “Eligibility criteria” (63.8%). The "funding" item received the lowest percentage (6.3%) of score 2.

### Risk of bias assessment

Only one study (0.8%) was assessed as having a low risk of bias, 75 (59%) RCTs were reported as “Some Concerns”, and 51 (40.2%) RCTs were identified as having a high risk of bias. Domain 5 (Selection of the reported results) of the RoB 2 tool raised some concerns in all the included RCTs (99.2%), except for one study, which received a low risk of bias rating. Although six studies were prospectively registered, five of them still exhibited “Some Concerns” in domain 5. These concerns stemmed from changes in the primary outcome for one study and insufficient information about the analysis, intervention groups, and discrepancies in the primary outcome time frame for four studies. The flaws identified in RCTs with a high risk of bias were typically found in domain 3 (bias due to missing outcome data) or/and domain 4 (bias in measurement of the outcome). We used a separate version of the RoB 2 tool for crossover studies for two of the included trials; one study received “Some Concerns”, and the other reported a high risk of bias for the extra domain “Domain s: Bias arising from period and carryover effects”. Nineteen of the 127 studies reported a low risk of bias for domains 1-4, except for domain 5, where discrepancies in the protocol or a lack of protocol registrations were observed. Domain 5 (Selection of the reported results) and domain 2 (Deviation from intended interventions) received the lowest percentage of low risk of bias ratings, with only 0.8% and 27.6%, respectively. The detailed assessment of the risk of bias for each included article, utilizing the RoB 2 tool, is presented in Supplementary file 4.

### Regression analysis of the variables

Table 2 presents the results of the unadjusted and multiple linear regression analyses. Multiple analyses revealed that older RCTs (both 6-10 years old and more than ten years old) had lower CONSORT scores (*p*<0.001) than more recent RCTs published within five years. Moreover, RCTs with a high risk of bias demonstrated lower CONSORT scores than those with low or some concerns. The RCTs published in journals that did not endorse the CONSORT statement within their author guidelines exhibited lower scores than those published in journals that endorsed the CONSORT statement. RCTs with unclear study designs and unclear funding sources were associated with a significant decrease in CONSORT scores compared to studies employing parallel or factorial designs and non-profit funding sources. RCTs with a registered protocol, whether prospective or retrospective, demonstrated higher CONSORT scores (*p*<0.001) than unregistered trials.

**Table 2:** Linear Regression between CONSORT scores and year science publication, RoB 2 overall assessment, journal endorsement of CONSORT, study design, funding, and protocol registration

Predictor Variables/Category	Unadjusted β^a^ (SE^b^)	P>|t|^*^	Adjusted β (SE)	P>|t|^*^
**Year Since Publication**				
≤5 years	Baseline		Baseline	
6-10 years	-7.98 (1.08)	<0.001*	-3.46 (0.83)	<0.001*
>10 years	-9.87 (1.20)	<0.001*	-5.04 (0.97)	<0.001*
**RoB 2 overall**				
Low or Some Concerns	Baseline		Baseline	
High	-4.52 (1.15)	<0.001*	-3.12 (0.63)	<0.001*
**Journal Endorsement of CONSORT**				
Yes	Baseline		Baseline	
No	-6.29 (1.19)	<0.001*	-2.75 (0.69)	<0.001*
**Study design**				
Parallel/factorial	Baseline		Baseline	
Split mouth/ crossover	-3.60 (1.75)	0.042*	-0.61 (1.01)	0.545
Unclear	-7.97 (1.48)	<0.001*	-3.33 (0.88)	<0.001*
**Funding**				
Non-Profit	Baseline		Baseline	
No Funding	5.41 (2.53)	0.035*	1.32 (1.43)	0.358
For-Profit	-1.23 (2.28)	0.591	-0.50 (1.28)	0.699
Unclear	-4.52 (1.22)	<0.001*	-2.81 (0.68)	<0.001*
**Protocol Registration**				
No Registration	Baseline		Baseline	
Prospective Registration	11.98 (2.03)	<0.001*	5.68 (1.53)	<0.001*
Retrospective Registration	10.87 (1.05)	<0.001*	5.31 (0.86)	<0.001*
**Country Income**				
High Income	Baseline			
Upper middle income	-1.32 (1.56)	0.401	-	-
Lower middle income or Low income	-2.42 (1.55)	0.122		
**Declaration to follow CONSORT**				
No	Baseline		-	-
Yes	5.97 (1.59)	<0.001*		
**H-index first author (Per unit)**	-0.10 (0.10)	0.307	-	-
**Impact Factor (2022)**	3.43 (1.16)	0.004*	-	-

^a^Coefficient Estimated

^b^Standard Error

^*^P < 0.05 considered of statistical significance

In the logistic regression analysis presented in Table 3, the adjusted results revealed that CONSORT scores within the range of 22-26 and scores ≥27 were significantly associated with 78% and 77% reduction in the odds of receiving high RoB2 ratings, respectively. Moreover, countries classified as upper-middle income had higher odds of receiving RoB2 ratings, indicating low or some concerns compared to high-income countries.

**Table 3:** Logistic regression analysis for the correlation between RoB 2 overall assessment and CONSORT overall scores, and country income

Predictor Variables/Category	Unadjusted Odds Ratio (95% CI^a^)	P>|z|^*^	Adjusted Odds Ratio (95% CI)	P>|t|^*^
**CONSORT scores**				
0-17	Baseline		Baseline	
18-21	0.96 (0.19-1.30)	0.440	0.79 (0.27-2.27)	0.659
22-26	0.29 (0.11-0.85)	0.022*	0.22 (0.07-0.67)	0.008*
≥27	0.24 (0.07-0.61)	0.007*	0.23 (0.08-0.69)	0.009*
**Country income**				
High income	Baseline		Baseline	
Upper middle income	0.34 (0.13-0.91)	0.031*	0.26 (0.09-0.74)	0.017*
Low middle income or low income	1.29 (0.52-3.21)	0.581	1.16 (0.43-3.17)	0.769
**Year Since Publication**				
≤5 years	Baseline			
6-10 years	1.13 (0.49-2.56)	0.779	-	-
>10 years	0.86 (0.34-2.17)	0.744		
**Study design**				
Parallel/factorial	Baseline			
Split mouth/ crossover	1.05 (0.30-3.70)	0.936	-	-
Unclear	2.20 (0.77-6.26)	0.139		
**Funding**				
Non-Profit	Baseline			
No Funding	1.39 (0.27-7.12)	0.691	-	-
For-Profit	6.50 (1.19-35.60)	0.031*		
Unclear	1.14 (0.51-2.55)	0.751		
**Protocol Registration**				
No Registration	Baseline			
Prospective Registration	0.62 (0.11-3.55)	0.590	-	-
Retrospective Registration	0.43 (0.17-1.12)	0.085		
**Declaration to follow CONSORT**			-	-
No	Baseline			
Yes	0.85 (0.31-2.33)	0.749		
**Journal Endorsement of CONSORT**			-	-
Yes	Baseline			
No	1.64 (0.75-3.55)	0.212		
**H-index first author (Per unit)**	0.95 (0.89-1.01)	0.131	-	-
**Impact Factor (2022)**	0.96 (0.47-1.96)	0.916	-	-

^a^95% CI= 95% Coefficient Interval

^*^P < 0.05 considered of statistical significance

**Fig. 3:**
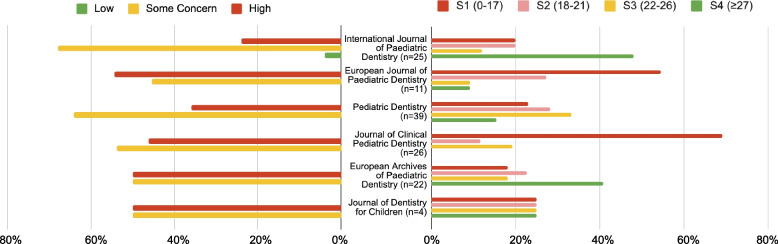
The percentages of overall CONSORT scores (S1= 0-17, S2= 18-21, S3=22-26, and S4= ≥27) and the Overall RoB 2 assessment (High, Some Concern, and Low) for each journal

Figure 3 provides an overview of each journal, displaying the percentages of overall CONSORT scores (S1= 0-17, S2= 18-21, S3=22-26, and S4= ≥27) and the overall RoB 2 assessment (High, Some Concern, and Low). The highest CONSORT overall score (36) was achieved by two RCTs [18, 19] published in the European Journal of Paediatric Dentistry and the International Journal of Paediatric Dentistry. These particular studies received favorable ratings with a low risk of bias in the first 4 domains, except in domain five, which was judged with some concerns.

## Discussion

Our study aimed to evaluate the adherence to the CONSORT checklist of RCTs addressing deep caries management published in six specific pediatric dental journals between 2010 and 2022. Our results revealed relatively low adherence to the CONSORT statement among RCTs focused on deep caries management in the selected pediatric dental journals from 2010 to 2022. This suggests ample room for improvement in transparency and reporting quality, and some improvement was observed in the last five years of our study. Nevertheless, the assessment of the risk of bias has uncovered methodologic flaws, with 126 studies raising concerns or displaying a high risk of bias. The findings suggest there is a potential risk that pediatric patients were exposed to experimental interventions with limited therapeutic benefits and possible adverse effects. Assessing the RCTs' internal validity and reporting quality is essential for determining their applicability in clinical practice. Research waste, defined as studies not benefiting society, is a major concern that can occur throughout the research process; evidence synthesis methods help identify low-priority research questions and avoid unnecessary studies [20]. Reducing research waste through evidence synthesis requires collaboration among investigators, publishers, and funding agencies.

Consistent with our findings, previous studies revealed low adherence to the CONSORT statement in various medical and dental fields. For instance, studies have shown suboptimal adherence in fields such as solid organ transplantation [21], pediatric dental journals [22], orthodontics [23], noncarious cervical lesions [13], endodontic regenerative procedures for necrotic immature teeth [24], posterior restorations [25], and dental bleaching [26]. One study [27], similar to ours, demonstrated an improvement in CONSORT adherence in recent years. The increase was relatively modest: for publications in the last five years, the improvement in CONSORT scores was only 3.46 (compared to those 6-10 years ago) and 4.04 points (compared to those more than ten years ago).

We observed a significant association between higher overall CONSORT scores and the endorsement of the CONSORT statement by journals. This highlights the positive impact of requiring authors to adhere to CONSORT checklists, which aligns with findings from previous studies [21, 28]. Nevertheless, there remains a need for attention from the editors and peer reviewers of all six included journals. While journals endorsing the CONSORT statement showed improvements in overall scores, a significant number of specific items still require attention. Therefore, it is suggested that journals not only endorse CONSORT but also engage in and provide improved training for editors, reviewers, and authors in the rigorous reporting of RCTs. Additionally, peer reviewers and editors should request authors to update the CONSORT item numbering after revisions and immediately before publication to ensure complete adherence to the reporting guidelines.

In contrast to an earlier study [27], which reported adequate documentation of items in RCTs on caries prevention, such as estimated effect size, protocol registration, trial design, outcomes, funding, title, and flow diagram, our findings indicate that these specific items were poorly reported in deep caries management trials. This finding aligns with previous studies [13, 25] that also highlighted these items for their inadequate reporting. Of particular concern was the insufficient description of sample size calculations, which is consistent with previous research [13, 26]. Explicitly stating sample size calculations is vital as it enhances predictability when interpreting intervention effects and bolsters the reproducibility of the RCT. These calculations are based on pre-specified estimates of the expected effect size for the primary outcome. This ensures that if no statistically significant group differences are found, it reflects a true absence of clinically meaningful effects rather than simply an underpowered study unable to detect important disparities.

The majority of the included studies, like previous research findings [13, 24, 25] did not adhere to the practice of pre-registering their trial protocols and subsequently disclosing this information within the published RCT. Trial protocol pre-registration ensures research transparency and prevents selective outcome reporting. This allows stakeholders to compare the published articles with the original planned protocol. Notably, only one study [29] in our analysis registered its trial protocol and followed the pre-registered protocol without any deviations, aligning with the CONSORT standards and the International Committee of Medical Journal Editors (ICMJE) [12, 30].

Most RCTs received poor scores for the “abstract” item, as they either intentionally or unintentionally omitted significant methodological details, such as type of study design, eligibility criteria, and statistical analysis methods. We underscore the importance of including the “title and abstract” items in the CONSORT tool assessment, as these sections are the most widely read and accessible parts of a research paper. Most readers form their initial judgments about a study based on these sections and quickly scan for key information to determine its relevance for further reading [31]. While word limits on abstracts, often imposed by journals, may inadvertently limit authors’ ability to include all pertinent details, this limitation does not justify incomplete communication of essential information. Despite these constraints, authors have an ethical obligation to transparently communicate crucial methodological details and primary results in the abstract. Further research is required to specifically evaluate the adherence of abstracts to the CONSORT abstract extension guidelines.

The items “allocation concealment” and “blinding” need further awareness, as they were either poorly reported or entirely omitted. In contrast, “sequence generation” received relatively good reporting, with approximately 67% of the included studies addressing the item adequately. Adequate reporting allocation concealment is needed, as it complements sequence generation by preventing knowledge of the sequence, ultimately reducing the risk of selection bias [13]. Allocation concealment should not be confused with blinding, as the former prevents selection bias, while the latter mitigates performance and detection bias [13]. Consistent with other studies [13, 23, 24], the most well-reported item was “Description of interventions”. This aspect is of particular significance, as it allows for the replication of procedures used to treat deep caries in children, facilitating the testing of their validity. Our risk of bias analysis revealed notable deficiencies in specific domains, namely, the randomization process, deviation from intended interventions, and selection of the reported results. This is in line with findings reported in previous studies [13, 24]. In our study, only 39.4% of the studies reported a low risk of bias for the domain “randomization process”, 27.6% reported a low risk of bias for the domain “deviation from intended interventions”, and the domain “selection of the reported results” reported a 0.8% low risk of bias. It is crucial to recognize that clinical trials categorized as having a significant risk of bias can substantially hinder our ability to draw reliable conclusions, potentially compromising the trustworthiness of caries management recommendations for clinical practice guideline developers. The deficiencies unveiled within RCTs highlight systemic issues in research methodology and transparency, exerting an impact on pediatric dental research. Identifying these common shortcomings paves the way for effective and cohesive quality improvement initiatives that may involve collaboration among editors, peer reviewers, and authors.

We emailed the authors of RCTs that did not report a protocol registration number in their manuscript. We requested that they provide us only with the registration number for their trial protocol if it was registered and did not ask them to provide any other information. This missing registry data affected our assessments of both the risk of bias using the RoB 2 tool and adherence to the CONSORT guidelines. For any other missing information regarding study design or methodology that was not declared by the authors in their manuscript, we did not contact them to request that information and considered it a deficiency in reporting quality, in line with the methodology employed by Loguercio et al. and Ortiz et al. [26, 27]. To maintain objectivity and avoid potential conflicts of interest arising from studies involving authors affiliated with this research, an external reviewer (MAH) with expertise in quality assessment was invited from another department. This reviewer conducted an independent evaluation alongside the other two reviewers. We limited our search to the specific journals chosen as they are well-established, peer-reviewed journals that publish research relevant to caries management topics in pediatric dentistry and that align with the methodology employed in previous empirical research to evaluate reporting quality [22, 32, 33]. Additionally, a key factor in their selection is that these six journals are indexed in the Web of Science. The use of the Web of Science allowed us to extract citation data for studies published in these journals, which was necessary for the analysis of this meta-research.

The adoption of the CONSORT statement by pediatric dental journals is a very important aspect and of great relevance for improving the reporting quality of RCTs of deep caries management in pediatric dental population. Our study underscores that adherence to the CONSORT statement remains relatively low, although improvements could be observed over the past 5 years. Furthermore, specific items highlighted in our study require further attention. This finding emphasizes the crucial role of the journal's active endorsement of the CONSORT, as shown previously [28]. As adherence to the CONSORT statement is associated with a reduced risk of bias, it is important to note that some concerns still exist within deep caries management RCTs in our target journals. Thus, clinical practice guideline developers and stakeholders still need to consider the results of the risk of bias analysis and evaluate the level of evidence included when formulating appropriate recommendations for clinicians.

## Conclusions

Our study highlights the imperative to enhance adherence to the CONSORT guideline and reduce the risk of bias in pediatric dentistry RCTs on deep caries management. This finding underscores the significance of endorsing the CONSORT statement by pediatric dental journals and emphasizes the necessity for authors, reviewers, and editors to evaluate manuscript compliance rigorously. Such measures are essential for improving the transparency and rigor of pediatric dental research. The study revealed deficiencies in critical areas, including prospective protocol registration, sample size estimation, and abstract reporting. These shortcomings demand attention and concerted efforts from all involved stakeholders.

### Supplementary Information

Supplementary Material 1.

Supplementary Material 2.

Supplementary Material 3.

Supplementary Material 4.

Supplementary Material 5.

## Data Availability

All data generated or analysed during this study are included in this published article [and its Supplementary information in Appendix S5 file]

## Abbreviations

RCTs: Randomized Controlled Trials
CONSORT: Consolidated Standards for Reporting of Trials
CPGs: Clinical Practice Guidelines
RoB: Risk of Bias
ICMJE: International Committee of Medical Journal Editors
