# Supplementary material for: CONSORT statement adherence and risk of bias in randomized controlled trials on deep caries management: a meta-research
Source: BMC Oral Health. 2024 Jun 13;24:687. doi: 10.1186/s12903-024-04417-0 (PMC11177528; doi:10.1186/s12903-024-04417-0)
Supplement: Supplementary file 1 — Supplementary Material 1. [file 12903_2024_4417_MOESM1_ESM.docx]

**Supplementary file 1:** PubMed/MEDLINE search strategy

| **Database** | **Search strategy, Limited to: 2010-2022** | **Results** |
| --- | --- | --- |
| PubMed/MEDLINE | ("International Journal of Paediatric Dentistry"[Journal] OR "European Journal of Paediatric Dentistry"[Journal] OR "PEDIATRIC DENTISTRY"[Journal] OR "The Journal of Clinical Pediatric Dentistry"[Journal] OR "Eur Arch Paediatr Dent"[Journal] OR "J Dent Child (Chic)"[journal]) AND ((randomizedcontrolledtrial[Filter]) AND (2010/1/1:2022/12/30[pdat])) | 458 |
