# Supplementary material for: CONSORT statement adherence and risk of bias in randomized controlled trials on deep caries management: a meta-research
Source: BMC Oral Health. 2024 Jun 13;24:687. doi: 10.1186/s12903-024-04417-0 (PMC11177528; doi:10.1186/s12903-024-04417-0)
Supplement: Supplementary file 2 — Supplementary Material 2. [file 12903_2024_4417_MOESM2_ESM.docx]

**Supplementary file 2:** The modified tool used to assess compliance of the studies with the CONSORT Statement

| CONSORT Item | Subitem | Score | Description |
| --- | --- | --- | --- |
| Title and abstract | Title | 0 | The information is not reported. |
|  |  | 1 | Incomplete information about the randomised trial in the title for example: clinical trial |
|  |  | 2 | Identification as a randomised trial in the title. |
|  | Abstract | 0 | The information is not reported. |
|  |  | 1 | Insufficient information about the topic or the methodological steps. |
|  |  | 2 | Structured summary of trial design, methods, results, and conclusions |
| Trial design |  | 0 | The information is not reported |
|  |  | 1 | 1. Information can be obtained by reading the manuscript, although the authors do not explicitly report it. 2. Consistence is lacking between sections of an article (e.g., abstract does not match the material and methods section; the presentation of the results does not match the description of the trial design; flow diagram presents different information, etc.). |
|  |  | 2 | The trial design is clearly written in the text (split mouth, parallel cross-over, factorial, or cluster). |
| Participants | Eligibility criteria | 0 | The information is not reported. |
|  |  | 1 | 1. Incomplete information of eligibility criteria compared to most of the studies in the field. 2. Presence of inconsistencies in the inclusion/exclusion criteria that prevent readers from knowing for which populations the intervention/control groups were performed. |
|  |  | 2 | The inclusion and exclusion criteria are clear so that readers can know exactly to which population the data can be extrapolated. |
|  | Setting and location | 0 | The setting and/or the location are not reported in the text. |
|  |  | 1 | 1. Authors describe either the setting or the date but never both. 2. This information can be obtained indirectly in the text. |
|  |  | 2 | Clear description of the setting (academic, practice-based research, university, private clinics, etc.) and the date when the intervention was implemented. |
| Intervention |  | 0 | No description is given. |
|  |  | 1 | Information is missing that prevents the replication of the interventions/comparators. |
|  |  | 2 | The interventions for each group are described with sufficient details to allow replication, including how they were actually administered. |
| Outcomes |  | 0 | No definition of the primary outcome and/or secondary outcomes is given. |
|  |  | 1 | 1. The authors only report they have used specific criteria without detailing the most important outcomes of such criteria. 2. The description of the primary outcome and/or secondary outcomes is very superficial and does not allow replication of the method. |
|  |  | 2 | At least the primary outcomes were defined in detail, including how and when they were assessed. Considered as clear when the details are clear, but the authors did not use the term ‘‘primary outcome’’ or related synonyms. |
| Sample size |  | 0 | No description is given in the article. |
|  |  | 1 | The sample size calculation is described but some parameters are missing so that it prevents replication. |
|  |  | 2 | Method of sample size calculation is described in a way that allows replication. The primary outcome for each sample size calculated should be identified. Elements of the sample size calculation for superiority trials is (1) the estimated outcomes in each group (which implies the clinically important target difference between the intervention groups); (2) the a (type I) error level; (3) the statistical power (or the "b [type II] error level); (4), for continuous outcomes, the standard deviation of the measurements should be reported. For equivalence trials, the equivalence limit instead of the effect size should be reported. |
| Randomisation | Sequence generation | 0 | No information is given in the text. |
|  |  | 1 | The authors only provide a very superficial description (such as the “groups were randomly allocated”) or do not provide sufficient information to allow replication of the randomisation  process. |
|  |  | 2 | 1. Clear description of the random sequence generation. 2. Or a clear description of a non-random sequence method. |
|  | Allocation concealment | 0 | No information is given in the text. |
|  |  | 1 | Partial reporting that prevents readers from fully replicating the method. |
|  |  | 2 | Clear description of the allocation concealment (such as sequentially numbered containers), fully described any steps taken to conceal the sequence until interventions. |
| Blinding |  | 0 | No description of the blinding is given. |
|  |  | 1 | Insufficient/partial information. For instance, (1) the authors describe examiners’ blinding or participants' blinding, but never both. 2. The authors describe the study as blind or double-blind but do not specify who was blinded. |
|  |  | 2 | 1. The authors describe who is blinded in the study. 2. In single-blind studies (when this is clearly reported by the authors), just the description of participant or evaluator (the one blinded) is enough; however, when a study is double blind or triple blind all blinded people should be described. 3. The study describes just the participant or examiner blinded but one of these participants cannot be blinded by intrinsic features of the study design. |
| Statistical methods | Hypothesis testing | 0 | Statistical methods are not described. |
|  |  | 1 | 1. Not enough information is given to evaluate the statistical method used by the authors, and/or the type of statistical tests employed by the authors are inadequate for the trial design and/or nature of the data (e.g., tests that do not considered the paired nature of the data when this is the case). 2. The authors describe several statistical tests but do not specify for each outcome they were applied. |
|  |  | 2 | Statistical methods are described with enough detail to enable a knowledgeable reader with access to the original data to verify the reported results. Additionally, statistical tests employed by the authors seem to be adequate for the type of trial design and nature of the data collected. |
|  | Estimated effect size | 0 | No descriptions of the effect size and 95% confidence interval are given. |
|  |  | 1 | Information is incomplete. |
|  |  | 2 | Authors report (at least for the primary outcome) the effect size and its precision (such as 95% confidence interval). Odds ratio, risk ratio, risk difference, mean difference, etc. are given. |
| Participant flow | Flow diagram | 0 | The flowchart is not presented in the article. |
|  |  | 1 | 1. Inconsistencies exist between the numbers described in the flowchart and other parts of the manuscript. 2. Incomplete diagram with missing information. |
|  |  | 2 | For each group, the numbers of participants who were randomly assigned, received intended treatment, and were analysed for the primary outcome is described in the flow chart CONSORT diagram. |
|  | Losses and Exclusions | 0 | No description of losses and exclusions is given. |
|  |  | 1 | Incomplete information. For instance, 1. The authors describe the overall percentage of losses, but this information is not specified per group, or 2. The authors describe the losses and exclusions but do not specify the reasons. |
|  |  | 2 | 1. For each group, losses and exclusions after randomization are described with reasons. 2.During reading, a reviewer can observe that no losses occurred to follow-up. |
| Baseline data |  | 0 | No table/text description with baseline data or description is given in the body of the text. |
|  |  | 1 | 1. A table/ text description with baseline data is presented but the data is not distributed between the study groups and/or it is given in percentages instead of raw numbers. 2. Insufficient information about participants/lesions is provided. 3. Inconsistencies in the data presented can be observed. |
|  |  | 2 | A table/text description containing baseline demographic and clinical characteristics of each group are presented in the article. |
| Numbers analysed |  | 0 | Authors do not report the numbers analysed. |
|  |  | 1 | No clear description of the number of participants (denominator) is included in the analysis of at least one of the outcomes. 2. Instead of reporting the raw number of participants, the authors report their data in percentages. 3. The authors fail to report the baseline number of patients included in each analysis. 4. Data can be obtained indirectly in the study. |
|  |  | 2 | For each group and for each outcome, the number or participants (denominator) included in the analysis is clear. |
| Registration and protocol |  | 0 | 1. This information is not available in the manuscript. 2. The authors considered that registration with an ethics committee is valid as a trial registry. 3.The authors describe that the study was registered but do not provide the registration number and/or the number provided does not link to the study. |
|  |  | 1 | The registry number was not declared inside the paper but obtained through contacting the corresponding author. |
|  |  | 2 | The study was registered in a trial registry and the protocol number is provided. |
| Funding |  | 0 | No information is given in the manuscript |
|  |  | 1 | 1.The authors describe that the study was funded without any information about the source of funding or the role of funders during the study. 2. The study was supported by a commercial company and the authors didn’t declare in the funding part or did not declare whether the funders had a role in conduction of the trial or not. |
|  |  | 2 | 1.The study reported the source of funding, type of funding (for-profit, non-profit or no funding) and the role of funders (i.e. the level of involvement by the funder or if they have no role the authors should state so. 2. the authors should inform if there is any other type of support such as supply and preparation of drugs or equipment, or in the analysis of data and writing of the manuscript. |
