# Supplementary material for: CONSORT statement adherence and risk of bias in randomized controlled trials on deep caries management: a meta-research
Source: BMC Oral Health. 2024 Jun 13;24:687. doi: 10.1186/s12903-024-04417-0 (PMC11177528; doi:10.1186/s12903-024-04417-0)

**Supplementary file 3**: The World Bank country income classification with the representative countries for each category and the percentages of the articles published between 2010-2022 in the target journals.


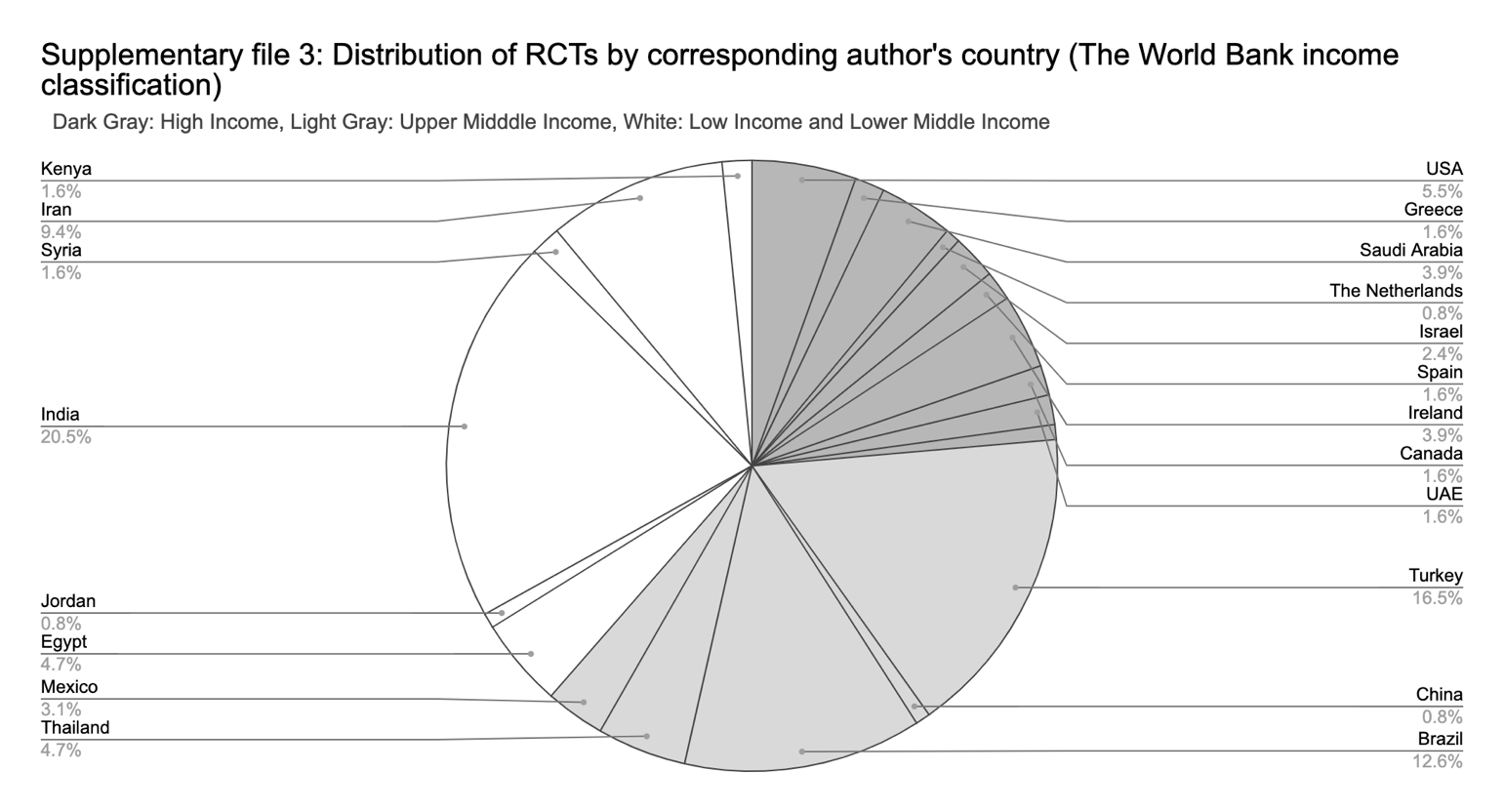

Supplement: Supplementary file 3 — Supplementary Material 3. [file 12903_2024_4417_MOESM3_ESM.docx]
