# Supplementary material for: CONSORT statement adherence and risk of bias in randomized controlled trials on deep caries management: a meta-research
Source: BMC Oral Health. 2024 Jun 13;24:687. doi: 10.1186/s12903-024-04417-0 (PMC11177528; doi:10.1186/s12903-024-04417-0)
Supplement: Supplementary file 4 — Supplementary Material 4. [file 12903_2024_4417_MOESM4_ESM.docx]

**Supplementary file 4, part 1:** The risk of bias assessment with RoB 2 tool for parallel RCTs.

| **PubMed ID** | **D1** | **D2** | **D3** | **D4** | **D5** | **Overall** |
| --- | --- | --- | --- | --- | --- | --- |
| 30345963 | L | SC | L | H | SC | H |
| 30788777 | L | SC | L | L | SC | SC |
| 30324561 | L | SC | L | H | SC | H |
| 31845435 | L | L | H | H | SC | H |
| 32185634 | SC | SC | L | L | SC | SC |
| 32278330 | L | L | L | L | SC | SC |
| 30992107 | L | SC | L | L | SC | SC |
| 22891625 | SC | SC | L | L | SC | SC |
| 32644890 | SC | SC | L | H | SC | H |
| 30702789 | L | L | L | L | SC | SC |
| 32644894 | SC | SC | L | L | SC | SC |
| 32516507 | L | L | L | L | SC | SC |
| 27759409 | SC | SC | H | L | SC | H |
| 30317420 | L | SC | L | L | SC | SC |
| 30888581 | L | L | H | H | SC | H |
| 22762173 | SC | SC | L | L | SC | SC |
| 23265154 | L | SC | H | L | SC | H |
| 25788172 | SC | SC | L | L | SC | SC |
| 20409203 | SC | SC | L | L | SC | SC |
| 27766712 | SC | L | L | L | SC | SC |
| 29380613 | L | L | L | L | SC | SC |
| 24683774 | SC | SC | H | L | SC | H |
| 26370384 | SC | L | H | L | SC | H |
| 30820833 | SC | SC | L | L | SC | SC |
| 24579274 | SC | SC | L | L | SC | SC |
| 24435546 | L | SC | H | L | SC | H |
| 31882030 | L | L | H | SC | SC | H |
| 23171351 | SC | SC | H | L | SC | H |
| 26370224 | L | L | L | L | SC | SC |
| 26161601 | SC | SC | L | L | SC | SC |
| 30864090 | SC | L | L | H | SC | H |
| 24960395 | SC | SC | L | L | SC | SC |
| 22838228 | SC | SC | L | H | SC | H |
| 28422591 | L | SC | L | L | SC | SC |
| 25102461 | SC | SC | H | L | SC | H |
| 22046692 | SC | SC | L | L | SC | SC |
| 33175326 | L | L | H | H | SC | H |
| 33543449 | L | L | L | L | SC | SC |
| 23270285 | SC | SC | H | L | SC | H |
| 27617373 | SC | SC | H | H | SC | H |
| 25951307 | SC | SC | L | H | SC | H |
| 24197970 | SC | SC | L | L | SC | SC |
| 30730794 | SC | L | L | L | SC | SC |
| 28292339 | L | L | L | L | SC | SC |
| 24351693 | L | SC | L | L | SC | SC |
| 27097854 | L | SC | L | L | SC | SC |
| 23855167 | SC | SC | L | L | SC | SC |
| 22502601 | SC | SC | H | L | SC | H |
| 28494595 | SC | SC | H | L | SC | H |
| 23756301 | SC | SC | H | L | SC | H |
| 22524073 | SC | SC | L | L | SC | SC |
| 26551364 | SC | SC | L | L | SC | SC |
| 22883359 | SC | SC | L | L | SC | SC |
| 236862855 | SC | SC | L | H | SC | H |
| 29070160 | L | L | L | L | SC | SC |
| 25631723 | SC | SC | L | L | SC | SC |
| 24011296 | SC | SC | H | L | SC | H |
| 23754205 | SC | SC | H | L | SC | H |
| 21070705 | SC | SC | L | L | SC | SC |
| 20836956 | SC | SC | L | L | SC | SC |
| 20642467 | SC | SC | L | L | SC | SC |
| 23346907 | SC | SC | L | L | SC | SC |
| 24164167 | SC | SC | H | H | SC | H |
| 22221174 | L | SC | L | L | SC | SC |
| 22211737 | L | SC | L | L | SC | SC |
| 34321144 | L | SC | H | H | L | H |
| 25102458 | SC | SC | H | H | SC | H |
| 29763354 | SC | L | L | L | SC | SC |
| 33719478 | L | L | L | L | SC | SC |
| 26370520 | SC | SC | L | L | SC | SC |
| 34192759 | SC | SC | L | L | SC | SC |
| 21902998 | SC | SC | L | L | SC | SC |
| 23534328 | SC | SC | H | H | SC | H |
| 20831133 | SC | SC | H | H | SC | H |
| 20642474 | L | SC | L | L | SC | SC |
| 21678668 | SC | SC | L | L | SC | SC |
| 33683572 | SC | L | H | L | SC | H |
| 22900437 | SC | SC | L | H | SC | H |
| 23211896 | SC | SC | H | L | SC | H |
| 23758458 | SC | SC | H | L | SC | H |
| 22077689 | SC | SC | L | L | SC | SC |
| 29767382 | SC | L | L | L | SC | SC |
| 22309243 | L | SC | L | L | SC | SC |
| 22046693 | SC | SC | L | H | SC | H |
| 26531075 | L | SC | H | L | SC | H |
| 21703078 | SC | SC | L | H | SC | H |
| 23597217 | SC | SC | L | L | SC | SC |
| 21883559 | SC | SC | H | L | SC | H |
| 26892210 | L | SC | L | L | SC | SC |
| 33728707 | L | L | L | H | SC | H |
| 30355428 | SC | SC | L | H | SC | H |
| 32522320 | L | SC | L | H | SC | H |
| 29122067 | SC | SC | L | L | SC | SC |
| 25197997 | L | SC | L | L | SC | SC |
| 29663908 | L | SC | L | L | SC | SC |
| 25514079 | SC | SC | H | L | SC | H |
| 20836951 | SC | SC | H | L | SC | H |
| 29070159 | L | L | L | L | SC | SC |
| 28292338 | L | SC | L | L | SC | SC |
| 21703067 | SC | SC | L | L | SC | SC |
| 21902995 | SC | SC | L | L | SC | SC |
| 23265162 | SC | SC | L | L | SC | SC |
| 28583242 | L | L | L | L | SC | SC |
| 22353416 | SC | SC | L | L | SC | SC |
| 29070158 | L | SC | L | L | SC | SC |
| 29793564 | L | L | L | L | SC | SC |
| 20298652 | SC | SC | L | L | SC | SC |
| 34740265 | SC | SC | L | H | SC | H |
| 34654496 | L | L | H | H | SC | H |
| 34937613 | L | L | L | L | SC | SC |
| 35986471 | L | L | L | L | SC | SC |
| 34783404 | L | L | L | L | SC | SC |
| 34856038 | L | L | L | L | L | L |
| 34398412 | SC | SC | H | L | SC | H |
| 35799339 | L | SC | L | L | SC | SC |
| 34138499 | L | L | L | L | SC | SC |
| 35751744 | SC | L | H | H | SC | H |
| 25197990 | SC | SC | H | L | SC | H |
| 23211895 | SC | SC | L | L | SC | SC |
| 23930631 | SC | SC | H | L | SC | H |
| 36098905 | L | L | L | L | SC | SC |
| 34599744 | L | L | L | L | SC | SC |
| 35152509 | L | L | L | L | SC | SC |
| 36099230 | SC | SC | L | H | SC | H |
| 35579583 | L | L | L | L | SC | SC |

**Part 2:** The risk of bias assessment with RoB 2 tool for crossover RCTs.

| **PubMed ID** | **D1** | **D2** | **D3** | **D4** | **D5** | **D6** | **Overall** |
| --- | --- | --- | --- | --- | --- | --- | --- |
| 25823480 | SC | H | SC | L | H | SC | H |
| 23342564 | SC | SC | SC | L | L | SC | SC |
